# Four novel species of *Pleurotheciaceae* collected from freshwater habitats in Jiangxi Province, China

**DOI:** 10.3389/fmicb.2024.1452499

**Published:** 2024-08-26

**Authors:** Wen-Ming He, Jun-Bo Zhang, Zhi-Jun Zhai, Danushka Sandaruwan Tennakoon, Chao-Yu Cui, Jian-Ping Zhou, Ming-Hui Chen, Hai-Jing Hu, Hua Yin, Yang Gao, Dian-Ming Hu, Hai-Yan Song

**Affiliations:** ^1^Bioengineering and Technological Research Center for Edible and Medicinal Fungi, Jiangxi Agricultural University, Nanchang, China; ^2^Nanchang Key Laboratory of Edible and Medicinal Fungi, Jiangxi Agricultural University, Nanchang, China; ^3^Jiangxi Key Laboratory for Excavation and Utilization of Agricultural Microorganisms, Jiangxi Agricultural University, Nanchang, China; ^4^Kunming Edible Fungi Institute of All China Federation of Supply and Marketing Cooperatives, Kunming, China; ^5^Key Laboratory of Crop Physiology, Ecology and Genetic Breeding, Jiangxi Agricultural University, Ministry of Education of the P.R. China, Nanchang, China

**Keywords:** diversity, four novel species, morphology, phylogeny, *Pleurotheciella*, *Pleurothecium*, taxonomy

## Abstract

During an investigation of fungal diversity from freshwater environments in different regions in Jiangxi Province, China, four interesting species were collected. Morphology coupled with combined gene analysis of an ITS, LSU, SSU, and *rpb2* DNA sequence data showed that they belong to the family *Pleurotheciaceae*. Four new species, *Pleurotheciella ganzhouensis*, *Pla. irregularis*, *Pla. verrucosa*, and *Pleurothecium jiangxiense* are herein described. *Pleurotheciella ganzhouensis* is characterized by its capsule-shaped conidia and short conidiophores, while *Pla. irregularis* has amorphous conidiophores and 3-septate conidia. *Pleurotheciella verrucosa* has cylindrical or verrucolose conidiogenous cells, 1-septate, narrowly fusiform, meniscus or subclavate conidia. *Pleurothecium jiangxiense* characterized in having conidiogenous cells with dense cylindrical denticles and short conidiophores. *Pleurothecium obovoideum* was transferred to *Neomonodictys* based on phylogenetic evidence. All species are compared with other similar species and comprehensive descriptions, micrographs, and phylogenetic data are provided.

## Introduction

Freshwater fungi refer to fungi that depend on aquatic environments for their entire or partial life cycle (Shearer, 1993; Calabon et al., 2023). They play a vital role in maintaining the balance of the freshwater ecosystem. Freshwater fungi are involved in the nutrient cycling of the ecosystem, they can decompose plant litter and other carbon sources that are difficult to degrade, such as insect bones, fish scales, and some animal hair (Palmer et al., 1997; Yuen et al., 1998; Vijaykrishna et al., 2005; Cai et al., 2006; Hyde et al., 2016). There have been some studies on lignicolous freshwater fungi in Jiangxi Province, China (Chen et al., 2022; Liu et al., 2022; Peng et al., 2022; Zhai et al., 2022; Yan et al., 2023). However, *Pleurotheciaceae* Réblová species have not been reported in Jiangxi Province so far.

*Pleurotheciaceae* was introduced by Réblová et al. (2015), and typified by *Pleurothecium*. Interestingly, most of *Pleurotheciaceae* species have been recorded from freshwater habitats, and currently eight genera are accepted, such as *Adelosphaeria* (Réblová et al., 2015), *Anapleurothecium* (Hernández-Restrepo et al., 2017), *Helicoascotaiwania* (Dayarathne et al., 2019), *Melanotrigonum* (Réblová et al., 2015), *Neomonodictys* (Hyde et al., 2020), *Pleurotheciella* (Réblová et al., 2012), *Pleurothecium* (Höhnel, 1919), and *Saprodesmium* (Dong et al., 2021). *Pleurotheciaceae* genera are highly varied, both morphologically and phylogenetically. Some are highly diverse with numerous species (e.g., *Pleurotheciella* and *Pleurothecium*). The species of *Pleurotheciella* and *Pleurothecium* are similar in having hyaline to brown macronematous conidiophores and denticulate conidiogenous cells (Luo et al., 2018; Hyde et al., 2020; Hyde et al., 2023). In addition, almost all of genera are monotypic and need more collections for their expansion (Luo et al., 2018; Réblová et al., 2020). Currently, there are nearly 60 species in *Pleurotheciaceae*, most of which are asexual morphs recorded from aquatic habitats (Luo et al., 2018).

*Pleurotheciella* was introduced by Réblová et al. (2012) to accommodate two species, *Pla. rivularia* and *Pla. centenaria*, which have nonstromatic peridium, unitunicate asci, persistent paraphyses and hyaline, and 3-septate ascospores. They have dactylaria-like asexual morph characterized by holoblastic, denticulate conidiogenous cells, subhyaline conidiophores and hyaline, septate conidia. Based on morphology and phylogenetic analyses, Réblová et al. (2015) transferred *Dactylaria uniseptata* Matsushima to *Pleurotheciella* as *Pla. uniseptata*. The species subsequently introduced later in *Pleurotheciella* are mainly asexual morphs. The morphological characteristics of most species in *Pleurotheciella* are similar to *Pleurothecium* in terms of conidiophores and denticulate conidiogenous cells, but they usually have conidia with a single septum (Hyde et al., 2018; Luo et al., 2018; Abdel-Aziz et al., 2020; Réblová et al., 2020; Dong et al., 2021; Shi et al., 2021). In this study, we introduce three new species of *Pleurotheciella* based on morphological characters and analyses of ITS, LSU, SSU, and rpb2 sequence data which are isolated from aquatic habitats in Jiangxi, China.

*Pleurothecium* is the type genus of *Pleurotheciaceae*, with *P. recurvatum* as the type species (Höhnel, 1919). This genus is characterized by distinct brown to light brown conidiophores, polyblastic sympodially extended denticulate conidiogenous cells, and solitary, 3-septate, hyaline or pigmented or bicolored conidia (Tubaki and Matsushima, 1972; Subramanian and Bhat, 1989; Cooper, 2005; Arzanlou et al., 2007; Yueming and Tianyu, 2009; Réblová et al., 2012; Monteiro et al., 2016; Hyde et al., 2017; Luo et al., 2018; Shi et al., 2021; Fryar and Catcheside, 2023; Jayawardena et al., 2023; Hyde et al., 2023). Currently, 16 species are accepted in this genus. In this study, we introduce a new *Pleurothecium* species based on morphological characters and analyses of ITS, LSU, SSU, and rpb2 sequence data, which is isolated from aqua tic habitats, in Jiangxi province, China.

In this study, we introduce four new species collected from Jiangxi province, China. Detailed descriptions and illustrations of morphological characteristics are provided for the new taxa.

## Materials and methods

### Samples collection, morphological observation, and isolation

Submerged decaying wood were collected from streams and rivers in Jiangxi province, China, and brought back to the laboratory in sealed plastic bags. The samples were incubated at room temperature (25°C) for 2 weeks in plastic boxes, spraying sterile water for moisturizing during the incubation. The samples were viewed under a Nikon SMZ-1270 microscope (Nikon Corporation, Japan) to observe fungi. Micro-morphological characteristics were observed and captured using a Nikon ECLIPSE Ni-U compound microscope (Nikon Corporation, Japan), equipped with a Nikon DS-Fi3 camera. All measurements were calculated using PhotoRuler Ver. 1.1 software.^1^ Figures were processed using Adobe Photoshop CS6 Extended version 10.0 software (Adobe Systems, United States) (Zhai et al., 2022).

Pure cultures of fungi were obtained by single spore isolation method described by Chomnunti et al. (2014). The germinated conidia were individually transferred to potato dextrose agar (PDA) and incubated at 25°C for 2 weeks. The fungal cultures were deposited in the Jiangxi Agricultural University Culture Collection (JAUCC) and the herbarium specimens were deposited in the Herbarium of Fungi Jiangxi Agricultural University (HFJAU).

### DNA extraction, PCR amplification, and sequencing

DNA was extracted from fresh mycelium on PDA using a modified cetyltrimethy lammonium bromide (CTAB) method (Doyle and Doyle, 1987). Four deoxyribonucleic acid (DNA) barcodes, ITS, LSU, SSU, and *rpb2*, were selected for polymerase chain reaction (PCR) using the primer pairs ITS1/ITS4 (White et al., 1990), LR0R/LR5 (Hopple and Vilgalys, 1999), NS1/NS4 (White et al., 1990), and fRPB2-5f/fRPB2-7cR (Liu et al., 1999), respectively. Amplification reactions were carried out in a volume of 25 μL, containing 12.5 μL 2 × Taq PCR MasterMix (Qingke, Changsha, China), 1 μL each forward and reverse primer (0.2 μM), 1 μL template DNA (*circa* 50–100 ng), and 9.5 μL ddH_2_O. Amplifications (ITS, LSU, and SSU) were conducted under the following conditions: 3 min at 98°C, 35 cycles of 10 s at 98°C, 10 s of annealing at 55°C and extension at 72°C for 10 s, with a final 2-min extension at 72°C. Regions of *rpb2* were amplified with initial denaturation of 95°C for 5 min, followed by 40 cycles of denaturation at 95°C for 1 min, annealing at 54°C for 90 s, elongation at 72°C for 90 s and the final extension at 72°C for 10 min included for each condition of amplification (Luo et al., 2018). Sequencing reactions were conducted with the corresponding forward and reverse primers commercially by QingKe Biotechnology Co. (Changsha, China). All sequences were edited with Sequencher v.4.14 (GeneCodes Corporation, United States) and have been deposited in the NCBI GenBank database (Table 1).

**Table 1:** Sequences used in this study.

Species	Collection/isolate number	ITS	LSU	SSU	*rpb2*	References
*Adelosphaeria catenata*	CBS 138679	NR_145396	NG_057081	NG_061211	KT278743	Réblová et al. (2015)
*Anapleurothecium botulisporum*	CBS 132713	NR_153582	-	-	-	Hernández-Restrepo et al. (2017)
*Dematipyriforma aquilariae*	MFLUCC:17-2382	OP377856	OP377941	OP378022	OP473101	Sun et al. (2017)
*Helicoascotaiwania farinosa*	DAOM 241947	JQ429145	JQ429230	-	-	Réblová et al. (2020)
*Helicoascotaiwania lacustris*	CBS 145964	MN699400	MN699431	MN699383	MN704305	Réblová et al. (2020)
	CBS 146144	MN699401	MN699432	MN699384	MN704306	Réblová et al. (2020)
	CBS 145963	MN699399	MN699430	MN699382	MN704304	Réblová et al. (2020)
*Melanotrigonum ovale*	CBS 138742	KT278723	KT278708	KT278695	KT278744	Réblová et al. (2015)
	CBS 138743	KT278724	KT278709	KT278696	KT278745	Réblová et al. (2015)
	CBS 138744	KT278725	KT278710	KT278697	KT278746	Réblová et al. (2015)
	CBS 138815	KT278722	KT278711	KT278698	KT278747	Réblová et al. (2015)
*Neomonodictys aquatica*	L-127	MZ686200	OK245417	-	-	Huang et al. (2022)
*Neomonodictys muriformis*	MFLUCC 16-1136	MN644509	MN644485	-	-	Hyde et al. (2020)
*Pleurotheciella ganzhouensis*	JAUCC6079	OR853417	OR853422	OR853426	PPO78759	This study
	JAUCC6678	PP180192	PP800214	PP801261	PP816289	This study
*Pleurotheciella irregularis*	JAUCC6080	OR853418	OR853423	PP801258	PP816286	This study
	JAUCC6679	PP180193	-	PP801262	-	This study
*Pleurotheciella verrucosa*	JAUCC6076	OR853414	OR853419	OR853424	PPO78756	This study
	JAUCC6675	PP800189	PP800211	PP801259	PP816287	This study
	JAUCC6078	OR853416	OR853421	PP801257	PPO78758	This study
	JAUCC6677	PP800191	PP800213	-	-	This study
*Pleurotheciella aquatica*	MFLUCC 17–0464	MF399236	MF399253	MF399220	MF401405	Luo et al. (2018)
*Pleurotheciella centenaria*	DAOM 229631	JQ429151	JQ429234	NG_064996	JQ429265	Réblová et al. 2012
*Pleurotheciella dimorphospora*	KUMCC 20-0185	NR_175737	NG_081519	NG_078760	-	Boonmee et al. (2021)
	MFLU 20-0138	MW981446	MW981444	MW981455	MZ509665	Boonmee et al. (2021)
*Pleurotheciella erumpens*	CBS 142447	NR_170010	MN699435	NG_070323	MN704311	…
*Pleurotheciella fusiformis*	MFLUCC 17–0115	MF399232	MF399249	MF399217	MF401402	Luo et al. (2018)
	MFLUCC 17–0113	MF399233	MF399250	MF399218	MF401403	Luo et al. (2018)
*Pleurotheciella guttulata*	KUMCC 15–0442	MF399239	MF399256	MF399222	MF401408	Luo et al. (2018)
	KUMCC 15–0296	MF399240	MF399257	MF399223	MF401409	Luo et al. (2018)
*Pleurotheciella krabiensis*	MFLU:18-0140	MG837018	MG837013	MG837023	-	Hyde et al. (2018)
*Pleurotheciella lunata*	MFLUCC 17–0111	MF399238	MF399255	MF399221	MF401407	Luo et al. (2018)
*Pleurotheciella rivularia*	CBS 125238	JQ429160	JQ429232	JQ429244	JQ429263	Réblová et al. (2012)
	CBS 125237	JQ429161	JQ429233	JQ429245	JQ429264	Réblová et al. (2012)
*Pleurotheciella saprophytica*	MFLUCC 16–1,251	MF399241	MF399258	MF399224	MF401410	Luo et al. (2018)
*Pleurotheciella submersa*	DLUCC 0739	MF399243	MF399260	MF399225	MF401411	Luo et al. (2018)
	MFLUCC 17–1709	MF399244	MF399261	MF399226	MF401412	Luo et al. (2018)
*Pleurotheciella sympodia*	MFLU 18-0995	MT555419	MT555425	MT555734	-	Shi et al. (2021)
	MFLUCC 18-0658	MT555418	MT559086	MT559094	-	Shi et al. (2021)
*Pleurotheciella tropica*	MFLU 18-0141	MG837020	MG837015	MG837025	-	Hyde et al. (2018)
*Pleurotheciella uniseptata*	S-936	MK878377	MK835846	MK834781	MN194025	Luo et al. (2018)
*Pleurothecium jiangxiense*	JAUCC6077	OR853415	OR853420	OR853425	PPO78757	This study
	JAUCC6676	PP800190	PP800212	PP801260	PP816288	This study
*Pleurothecium aquaticum*	MFLU:21-0148	OM654775	OM654772	OM654807	OM672034	Luo et al. (2018)
*Pleurothecium aquisubtropicum*	GZCC 21–0670	OM339436	OM339433	-	-	Jayawardena et al. (2023)
*Pleurothecium brunius*	SCF-2023a	OQ799378	OQ799377	OQ799376	-	Fryar and Catcheside (2023)
*Pleurothecium floriforme*	MFLUCC 15-0628	KY697281	KY697277	NG_063634	-	Hyde et al. (2017)
*Pleurothecium guttulatum*	IFRD 9203	MT555415	MT559115	MT559089	-	Shi et al. (2021)
*Pleurothecium hainanense*	GZCC 22-2021	OP748934	OP748931	-	-	Hyde et al. (2023)
*Pleurothecium obovoideum*	CBS 209.95*	EU041784	EU041841	-	-	Arzanlou et al. (2007)
*Pleurothecium pulneyense*	MFLUCC 16-1293	-	MF399262	MF399228	MF401414	Luo et al. (2018)
*Pleurothecium recurvatum*	CBS 131272	JQ429149	JQ429237	JQ429251	JQ429268	Réblová et al. (2012)
	CBS 131646	JQ429150	JQ429236	JQ429250	-	Réblová et al. (2012)
*Pleurothecium semifecundum*	CBS 131271	JQ429159	JQ429240	NG_062854	JQ429270	Réblová et al. (2012)
*Saprodesmium dematiosporum*	KUMCC 18-0059	MW981646	MW981647	NG_148854	-	Dong et al. (2021)
*Sterigmatobotrys macrocarpus*	PRM 915682	JQ429153	GU017317	-	-	Réblová et al. (2012)
*Sterigmatobotrys rudis*	DAOM 229838	JQ429152	JQ429241	JQ429256	JQ429272	Ertz et al. (2016)

### Phylogenetic analyses

The combined dataset consists of 39 taxa including our newly generated taxa. *Dematipyriforma aquilariae* was used as the out-group taxon. Taxa with the highest similarities to our strains were determined with standard nucleotide BLASTn searches in GenBank.^2^ The other sequences used in the analyses were obtained from the recent publications (Luo et al., 2018; Réblová et al., 2020). Detailed information on fungal strains used in this paper is provided in Table 1.

All obtained sequences were aligned using the online service of MAFFT (Madeira et al., 2019) and refined manually in MEGA v.7.0 (Kumar et al., 2016). Maximum likelihood (ML) analysis was conducted using RAxML 8.0 with GTR-GAMMA model of evolution (Stamatakis, 2014). Non-parametric bootstrap analysis was implemented using 1,000 replicates to estimate ML bootstrap (BS) values. Bayesian Inference analysis was carried out with PhyloSuite_v1.2.2_Win under partitioned models (Ronquist et al., 2012). The best-fit models of nucleotide substitutions were selected according to the Bayesian Information Criterion (BIC) implemented in ModelFinder on PhyloSuite_v1.2.2_Win. The models for ITS(1–599), LSU(600–1,458), SSU(1,459–2,432), and *rpb2*(2,433–3,322) datasets used for phylogenetic analysis are GTR + F + I + G4 model, SYM + I + G4 model, SYM + I + G4 model, and GTR + F + I + G4 model, respectively. The datasets were run for 10,000,000 generations, with four chains and trees sampled every 1,000th generation. The first 10% trees were discarded as burn-in. The Bayesian consensus tree with posterior probabilities (PP) was visualized with FigTree v.1.4.4 (Rambaut, 2018) and was edited in Adobe Illustrator CS6.

## Results

### Phylogenetic results

According to the BLAST results, the ITS sequence of *Pleurothecium jiangxiense* shares 90.3% similarity to *Pleurothecium brunius* with 51 nucleotide differences (18 gaps). The ITS of *Pla. verrucosa* shares 93.81% similarity to *Pla. Krabiensis* with 33 nucleotide differences (four gaps). The ITS sequence of *Pla. ganzhouensis* shares 95% similarity (25 nucleotide differences, of which 18 are gaps) with that of *Pla. rivularia*. In addition, *Pla. irregularis* shares 98.8% similarity (six nucleotide differences) to *Pla. centenaria*.

**Figure 1:**
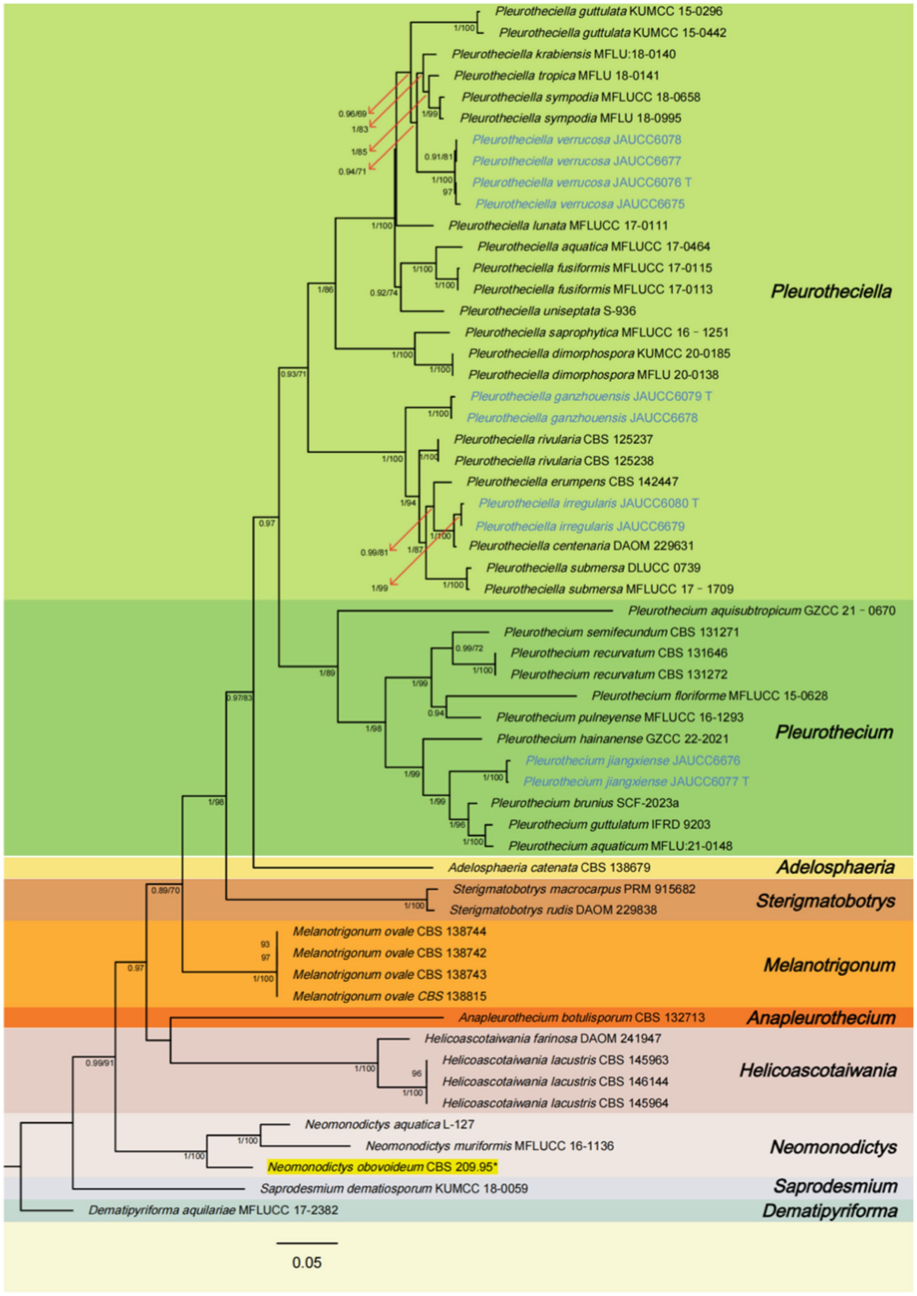
Phylogenetic tree of *Pleurotheciaceae*, inferred from the combined regions (ITS-LSU-SSU-rpb2) using Bayesian Inference (BI) analysis. The *Dematipyriforma aquilariae* was used as the outgroup. The lineages with new species were shown in blue font. The lineages with adjusted species were shown in yellow background. PP ≥ 0.90 and BS ≥ 70% were indicated around the branches. Supported clade (PP/BS = 1.00/99) with the lineage consisting of *Pleurothecium brunius*, *Pleurothecium aquaticum*, and *Pleurothecium guttulatum*. Species of *Saprodesmium* and *Neomonodictys* have longer genetic distance from other species of *Pleurotheciaceae*. *Sterigmatobotrys* groups together with species of *Pleurotheciaceae* with a strong statistical support.

The aligned matrix for the combined analysis, ITS + LSU + SSU + rpb2, comprised 3,322 bp, including 599 bp of ITS, 859 bp of LSU, 974 bp of SSU, and 890 bp of rpb2. The combined ITS, LSU, SSU, and rpb2 dataset consisted of 57 sequences representing 36 species of the *Pleurotheciaceae*, two sequences representing two species of *Sterigmatobotrys*, and one sequence representing one species of *Dematipyriforma*. The topologies of trees generated by ML and BI analyses are congruent. The Bayesian tree with BS and PP is shown in Figure 1.

All species of *Pleurotheciaceae* form a monophyletic group. *Pleurotheciella verrucosa* groups together with *Pla. krabiensis*, *Pla. tropica*, and *Pla. sympodia* (PP/BS = 0.94/71), which four collections are from two different regional freshwater habitats with a strong-supported clade (BS/PP = 100/1.00). The strains of *Pleurotheciella ganzhouensis* form a strong-supported clade (PP/BS = 1.00/100), group together with *Pla. rivularia*, *Pla. erumpens*, *Pla. irregularis*, *Pla. centenaria*, and *Pla. submersa*. Collections of *Pleurotheciella irregularis* form a strong-supported clade (PP/BS = 1.00/99) with *Pla. centenaria*. Collections of *Pleurothecium jiangxiense* form a strongly supported clade (PP/BS = 1.00/99) with the lineage consisting of *P. brunius*, *P. aquaticum*, and *P. guttulatum*. Species of *Saprodesmium* and *Neomonodictys* have longer genetic distance from other species of *Pleurotheciaceae*. *Sterigmatobotrys* groups together with species of *Pleurotheciaceae* with a strong statistical support.

### Taxonomy

***Pleurotheciella ganzhouensis* W.M. He, D.M. Hu & H.Y. Song, sp. nov.**

**MycoBank number:** MB853181

**Typification:** Qing Tang Zhen 515 Xiang Dao Xie Cun, Ningdu County, Ganzhou City, Jiangxi Province, China (江西省赣州市宁都县青塘镇515乡道谢村). Longitude: E115.562124° Latitude: N26.404602°, on 30 Jan 2023, W.M. He, (HFJAU 10280, holotype), ex-type culture JAUCC6079. **Additional specimen examined:** Qing Tang Zhen 515 Xiang Dao Xie Cun, Ningdu County, Ganzhou City, Jiangxi Province, China (江西省赣州市宁都县青塘镇515乡道谢村). Longitude: E115.562124° Latitude: N26.404602°, on 5 Jun 2023, W.M. He, (HFJAU 10413, paratype), ex-paratype culture JAUCC6678. **GenBank number:** JAUCC6079: ITS = OR853417, LSU = OR853422, SSU = OR853426, rpb2 = PPO78759. JAUCC6678: ITS = PP180192, LSU = PP800214, SSU = PP801261, rpb2 = PP816289.

**Etymology:** Referring to the collecting place, Ganzhou city, Jiangxi Province, China.

*Saprobic* on submerged decaying wood. **Sexual morph:** Undetermined. **Asexual morph:** *Colonies* grow on the wood surface, white, reflective, clustered, upper part covered with bright white mass of conidia, outward radially distributed. *Mycelium* immersed, partly superficial, relatively sparse, composed with unbranched, hyaline and 1.4–3.2 μm wide hyphae, with few conidiophores on the superficial part. *Conidiophores* 9.9–41.9 μm (−*x*  = 16.4 μm, *n* = 30) long, 2.2–3.7 μm (^−*x* ^ = 2.9 μm, *n* = 30) wide, macronematous, mononematous, 1-septate or aseptate, smooth, cylindrical, hyaline, erect or slightly curved, the top slightly swollen with denticulate conidiogenous sites. Denticulate. *Conidiogenous* cells integrated, terminal, polyblastic, cylindrical or verrucous, hyaline, forming conidia sympodially on cylindrical denticles. *Conidia* 14.4–19.4 (−*x*  = 17.1, *n* = 30) × 2.5–3.3 (−*x*  = 2.9, *n* = 30) μm, capsule-shaped, fusiform, cylindrical or subclavate, hyaline, guttulate, straight or slightly arcuate, 1-septate, round and tapering at both ends, one end is usually sharper, smooth-walled.

**Figure 2:**
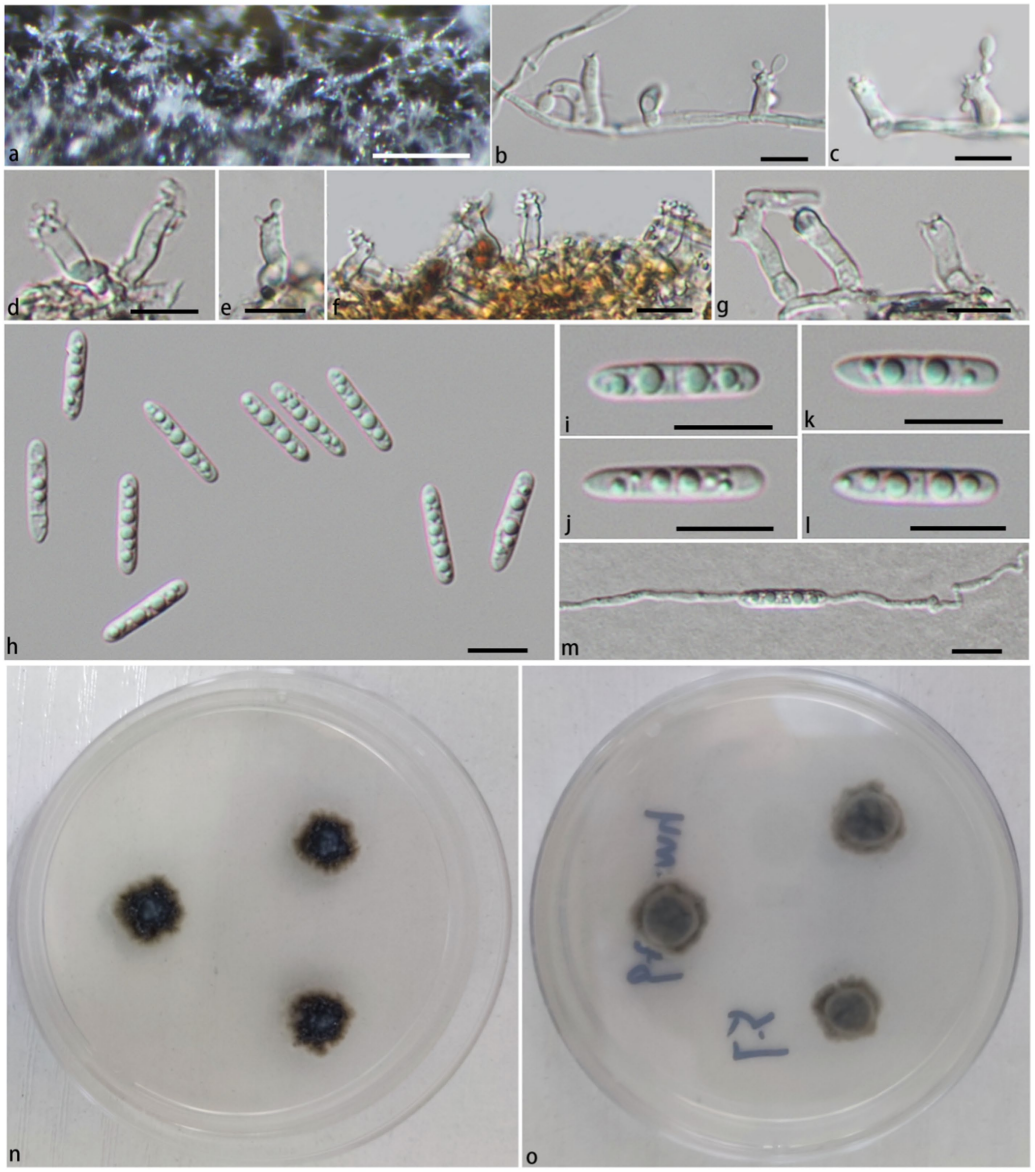
*Pleurotheciella ganzhouensis* (HFJAU 10280, holotype). **(A)** Colonies on submerged wood. **(B–G)** Conidiophores and conidiogenous cells. **(H–L)** Conidia. **(M)** A germinating conidium. **(N,O)** Cultures on PDA medium (**N** from above, **O** from below). Scale bars: **(A)**= 100 μm. **(B–M)** = 10 μm.

In the phylogenetic tree (Figure 1), *Pla. ganzhouensis* formed an independent lineage sister to a clade containing *Pla. rivularia*, *Pla. erumpens*, *Pla. centenaria*, *Pla. amorphous*, and *Pla. submersa* (PP/BS = 1.00/100). Morphologically, compared with other species of *Pleurotheciella*, *Pla. ganzhouensis* have unique capsule-shaped conidia and shorter conidiophores (9.9–41.9 × 2.2–3.7 μm), and with few hyaline conidiophores on superficial part of mycelium, which can be clearly distinguished from other species of *Pleurotheciella* (Hyde et al., 2018; Luo et al., 2018; Abdel-Aziz et al., 2020; Réblová et al., 2020; Dong et al., 2021; Shi et al., 2021). In addition, combined with its short conidiophores, it is similar to the species of *Neta*, but its 1-septate, slender and capsule-shaped conidia can be clearly distinguished from *Neta* (Figure 2).

***Pleurotheciella irregularis* W.M. He, D.M. Hu & H.Y. Song, sp. nov.**

**MycoBank number:** MB853182

**Material examined:** Shenlong Tan, Taiping town, Xinjian District, Nanchang City, Jiangxi Province, China (江西省南昌市新建区太平镇神龙潭). Longitude: 115.697°E Latitude: 28.766°N, on 19 Mar 2023, W.M. He, (HFJAU 10281, holotype), ex-type culture JAUCC6080. **Additional specimen examined:** Shenlong Tan, Taiping town, Xinjian District, Nanchang City, Jiangxi Province, China (江西省南昌市新建区太平镇神龙潭). Longitude: 115.697°E Latitude: 28.766°N, on 7 Oct 2023, W.M. He, (HFJAU 10414, paratype), ex-paratype culture JAUCC6679. **GenBank number:** JAUCC6080: ITS = OR853418, LSU = OR853423, SSU = PP801258, rpb2 = PP816286. JAUCC6679: ITS = PP180193, SSU = PP801262.

**Etymology:** Refers to the irregular shape of conidiophores.

*Saprobic* on submerged decaying wood. **Sexual morph:** Undetermined. **Asexual morph:** *Colonies* grow on the submerged wood surface, white, reflective, clustered, radiant growth from the bottom, upper part covered with bright white mass of conidia. *Mycelium* immersed, relatively dense, brown, composed with 2.3–3.5 μm wide, unbranched hyphae. *Conidiophores* 50–110 μm long, 2.6–3.5 μm wide, macronematous, mononematous, mostly curved, hyaline, aseptate, verrucous, irregular, cylindrical mostly tapering toward the apex, some with a terminal node of denticles, with verruca in the middle and upper part. *Conidiogenous cells* integrated, terminal, polyblastic, cylindrical or verrucous, hyaline forming conidia sympodially on cylindrical denticles. *Conidia* 24.2–33.9 μm (−*x* = 29.4 μm, *n* = 30) × 4.2–6.4 μm (−*x*  = 5.4 μm, *n* = 30), narrowly fusiform, subclavate, hyaline, guttulate, straight or slightly arcuate, 1-3-septate, slightly constricted at the septum, pointed at one end the other round an wider in the middle, smooth-walled.

**Figure 3:**
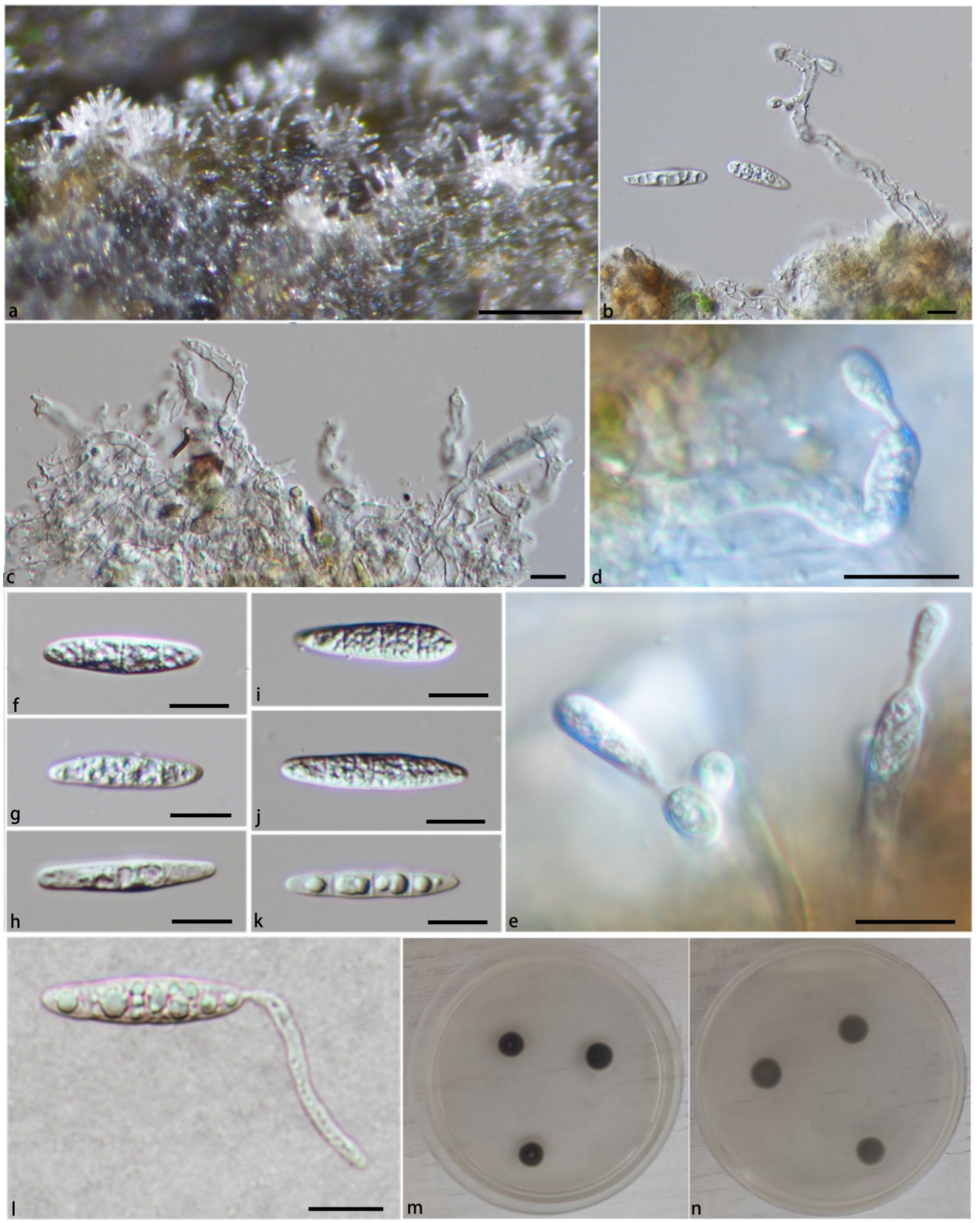
*Pleurotheciella irregularis* (HFJAU 10281, holotype). **(A)** Colonies on submerged wood. **(B,C)** Conidiophores and conidia. **(D,E)** Conidiophores and conidiogenous cells bearing conidia. **(F–K)** Conidia. **(L)** A germinating conidium (**M** from above, **N** from below). **(M,N)** Culture on PDA. Scale bars: **(A)** = 100 μm; **(B–L)** = 10 μm.

**Notes:** In the phylogenetic tree (Figure 1), *Pla. irregularis* form a strongly supported clade (PP/BS = 1.00/100) with the strains of *Pla. centenaria*. Morphologically, *Pla. irregularis* is unique in its amorphous conidiophores. In addition, our collection can be distinguished from *Pla. centenaria* by larger conidia (24.2–33.9 × 4.2–6.4 μm vs. 18.0–22.5 × 4.0–5.5 μm) and longer conidiophores (50–110 × 2.6–3.5 μm vs. 12.0–35.0 × 3.0–4.5 μm) (Réblová et al., 2012) (Figure 3).

***Pleurotheciella verrucosa* W.M. He, D.M. Hu & H.Y. Song, sp. nov.**

**MycoBank number:** MB853180

**Typification:** Tang long, 824 County highway, Wanan County, Jian City, Jiangxi Province (江西省吉安市万安县824县道塘咙). Longitude: E115.029429° Latitude: N26.224633°, on 30 Jun 2022, W.M. He (HFJAU 10277, holotype), ex-type culture JAUCC6076, ex-paratype culture JAUCC6675. **Additional specimen examined:** Tang long, 824 County highway, Wanan County, Jian City, Jiangxi Province (江西省吉安市万安县824县道塘咙). Longitude: E115.029429° Latitude: N26.224633°, on 3 October 2022, W.M. He (HFJAU 10410, paratype), ex-paratype culture JAUCC6675. Qing Tang Zhen 515 Xiang Dao Xie Cun, Ningdu County, Ganzhou City, Jiangxi Province, China (江西省赣州市宁都县青塘镇515乡道谢村). Longitude: E115.562124° Latitude: N26.404602°, on 30 Jan 2023, W.M. He, (HFJAU 10279, paratype), ex-paratype culture JAUCC6078. Qing Tang Zhen 515 Xiang Dao Xie Cun, Ningdu County, Ganzhou City, Jiangxi Province, China (江西省赣州市宁都县青塘镇515乡道谢村). Longitude: E115.562124° Latitude: N26.404602°, on 5 Jun 2023, W.M. He, (HFJAU 10412, paratype), ex-paratype culture JAUCC6677. **GenBank number:** JAUCC6076: ITS = OR853414, LSU = OR853419, SSU = OR853424, rpb2 = PPO78756. JAUCC6675: ITS = PP800189, LSU = PP800211, SSU = PP801259, rpb2 = PP816287. JAUCC6078: ITS = OR853416, LSU = OR853421, SSU = PP801257, rpb2 = PPO78758. JAUCC6677: ITS = PP800191, LSU = PP800213.

**Etymology:** Referring to apical conidiophores with verrucose conidiogenous warts.

*Saprobic* on submerged decaying wood. **Sexual morph:** Undetermined. **Asexual morph:** *Colonies* grow on the surface of wood, light brown, reflective, clustered, most upright, upper part covered with bright white mass of conidia. *Mycelium* 1.2–2.2 μm wide, immersed, relatively sparse, light brown, with few or no branches. *Conidiophores* 51.3–131.8 μm (−*x* = 89.4 μm, *n* = 60) long, 1.9–3.4 μm (−*x* = 2.62 μm, *n* = 60) wide, macronematous, mononematous, septate, smooth, cylindrical, dark brown at the base, becoming paler toward the apex, erect or slightly curved, bottom slightly swollen, *Conidiogenous cells* integrated, terminal, polyblastic, cylindrical or verrucolose, pale brown to hyaline, forming conidia sympodially on cylindrical denticles or wart. *Conidia* 10.2–16.9 μm (−*x* = 14.1 μm, *n* = 60) × 2.3–4.3 μm (−*x* = 3.4 μm, *n* = 60), narrowly fusiform, meniscus or subclavate, hyaline, guttulate, straight or arcuate, uniseptate, pointed at one end, the other round and wide in the middle smooth-walled.

**Figure 4:**
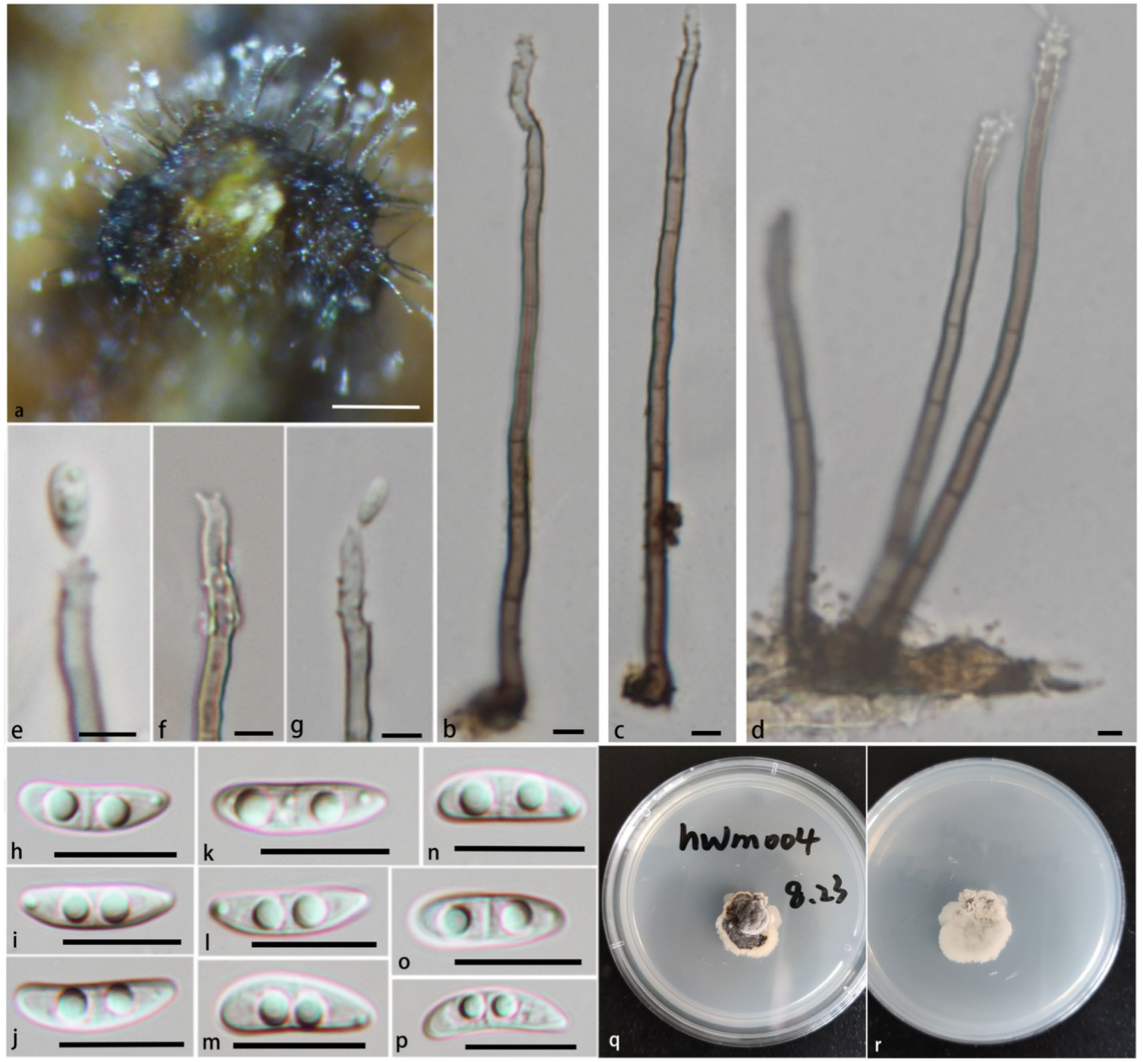
*Pleurotheciella verrucosa* (HFJAU 10277, holotype). **(A)** Colonies on submerged wood. **(B–G)** Conidia and conidiogenous cells. **(H–P)** Conidia. **(Q,R)** Cultures on PDA medium (**Q** from above, **R** from below). Scale bars: **(A,B)** = 100 μm. **(B–G)** = 5 μm. **(H–P)** = 10 μm.

**Figure 5:**
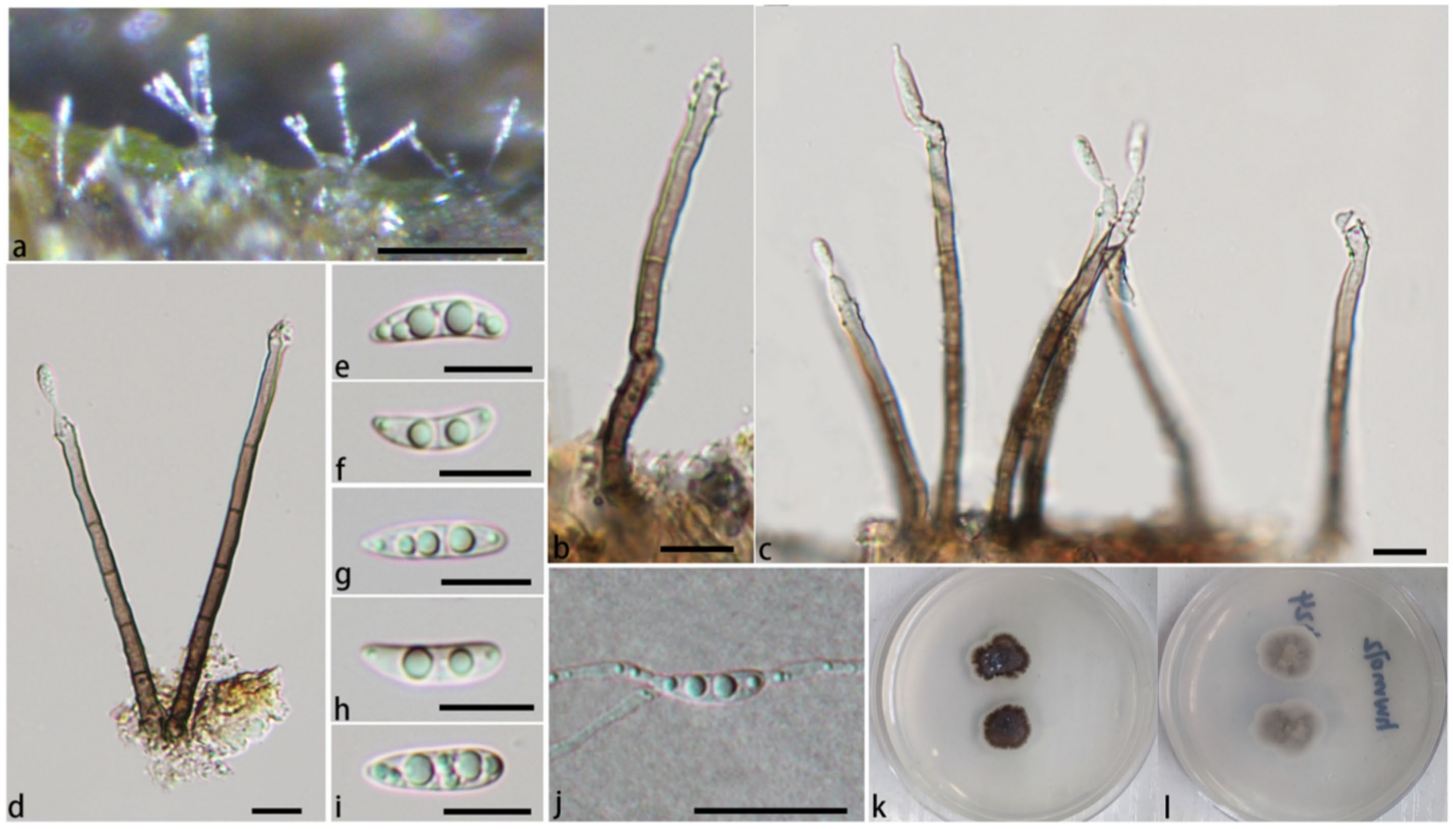
*Pleurotheciella verrucosa* (HFJAU 10279). **(A)** Colonies on submerged wood. **(B–D)** Conidiophores and conidiogenous cells. **(E–I)** Conidia. **(J)** A germinating conidium. **(K,L)** Cultures on PDA medium (**K** from above, **L** from below). Scale bars: **(A)** = 100 μm. **(B–J)** = 10 μm.

**Notes:** In the phylogenetic tree (Figure 1), *Pla. verrucosa* groups together with a clade containing *Pla. krabiensis*, *Pla. tropica*, and *Pla. sympodia* with moderate statistical support (PP/BS = 0.94/71). Morphologically, *Pla. verrucosa* is easily distinguished from *Pla. krabiensis* by its shorter and finer conidiophores (51.3–131.8 × 1.9–3.4 μm vs. 240–390 × 3.3–4.8 μm) and smaller conidia (10.2–16.9 × 2.3–4.3 μm vs. 19–25 × 4.5–6 μm) (Hyde et al., 2018). *Pleurotheciella tropica* differs from *Pla. verrucosa* in having longer and wider conidiophores (100–250 × 4–4.8 μm vs. 51.3–131.8 × 1.9–3.4 μm) and bigger conidia (16–21 × 5.5–7 μm vs. 10.2–16.9 × 2.3–4.3 μm) (Hyde et al., 2018). *Pleurotheciella sympodia* differs from *Pleurotheciella verrucosa* in having longer conidiophores (135–355 × 1.5–3.5 μm vs. 51.3–131.8 × 1.9–3.4 μm) and larger conidia (22.5–29 × 4.5–6.5 μm vs. 10.2–16.9 × 2.3–4.3 μm) (Shi et al., 2021) (Figures 4, 5).

***Pleurothecium jiangxiense* W.M. He, D.M. Hu & H.Y. Song, sp. nov.**

**MycoBank number:** MB853179

**Typification:** Shunfeng village, Wanan country, Jian city, Jiangxi province, China (江西省吉安市万安县顺峰乡). Longitude: E115.015900° Latitude: N26.198737°, on 30 Jun 2022, W.M. He, (HFJAU 10278, holotype), ex-type culture JAUCC6077. **Additional specimen examined:** Shunfeng village, Wanan country, Jian city, Jiangxi province, China (江西省吉安市万安县顺峰乡). Longitude: E115.015900° Latitude: N26.198737°, on 3 Oct 2022, W.M. He, (HFJAU 10411, paratype), ex-paratype culture JAUCC6676. **GenBank number:** JAUCC6077: ITS = OR853415, LSU = OR853420, SSU = OR853425, *rpb2* = PPO78757. JAUCC6676: ITS = PP800190, LSU = PP800212, SSU = PP801260, *rpb2* = PP816288.

**Etymology:** Referring to the collecting place, Jiangxi Province, China.

*Saprobic* on submerged decaying wood. **Sexual morph:** Undetermined. **Asexual morph:** *Colonies* on the substratum superficial, effuse, solitary, shiny, white to hyaline. *Mycelium* 1–1.5 μm wide, composed of partly immersed, partly superficial, light brown to hyaline, septate, unbranched hyphae, superficial part differentiating into conidiogenous cells, with some conidia. *Conidiophores* 6.2–17.8 μm (−*x* = 11.8 μm, *n* = 15) long, 2.2–5.3 μm (−*x* = 3.4 μm, *n* = 15) wide, mononematous, cylindrical, unbranched, aseptate, straight or slightly curved, light brown to hyaline, rough-walled. *Conidiogenous cells* 2.6–8.4 μm long, 2.6–4 μm diam, integrated, holoblastic, polyblastic, ellipsoidal or cylindrical, hyaline to light brown, distributed at the middle and apex of the conidiophores, conidiogenous loci denticulate, denticles discrete, determinate, 1.8–4.2 × 0.4–0.6 μm. *Conidia* 15.1–20.5 μm (−*x* = 17.5 μm, *n* = 30) × 4.3–6.2 μm (−*x* = 5.3 μm, *n* = 30), acrogenous, holoblastic, grow on denticles, hyaline, 3-septate, guttulate, slightly curved, subclavate, cylindrical, ellipsoidal, rounded at both ends, conidial secession schizolytic, smooth-walled.

**Notes:** In the phylogenetic analysis (Figure 1), *P. jiangxiense* clades with *P. aquaticum*, *P. brunius*, and *P. guttulatum* with strong statistical support (PP/BS = 1.00/99), then clades with *P. hainanense* with strong statistical support (PP/BS = 1.00/99).

Morphologically, the conidiophores of *P. jiangxiense* are significantly short (6.2–17.8 × 2.2–5.3 μm), which are significantly different from other species of the genus. *P. jiangxiense* have obvious superficial hyphae between conidiophores, which can differentiate into conidiogenous cells and with some conidia, this phenomenon has not been observed in other species of the genus (Tubaki and Matsushima, 1972; Subramanian and Bhat, 1989; Cooper, 2005; Arzanlou et al., 2007; Yueming and Tianyu, 2009; Réblová et al., 2012; Monteiro et al., 2016; Hyde et al., 2017; Luo et al., 2018; Shi et al., 2021; Fryar and Catcheside, 2023; Jayawardena et al., 2023; Hyde et al., 2023). Compare with *P. brunius*, *P. aquaticum* and *P. guttulatum*, conidia of *P. jiangxiense* have more rounded bottom, and not constricted at the septa. The size of the conidia of *P. jiangxiense* is shorter than *P. aquaticum* (15.1–20.5 × 4.3–6.2 μm vs. 19–21 × 4.5–5.5 μm), *P. guttulatum* (15.1–20.5 × 4.3–6.2 μm vs. 22–28 × 5–6 μm), and *P. yunnanensis* (15.1–20.5 × 4.3–6.2 μm vs. 17–25.6 × 2.8–9 μm), almost similar to *P. brunius* (15.1–20.5 × 4.3–6.2 μm vs. 16–19 × 5–6 μm) (Luo et al., 2018; Shi et al., 2021; Fryar and Catcheside, 2023; Chun-Sheng et al., 2023).

**Figure 6:**
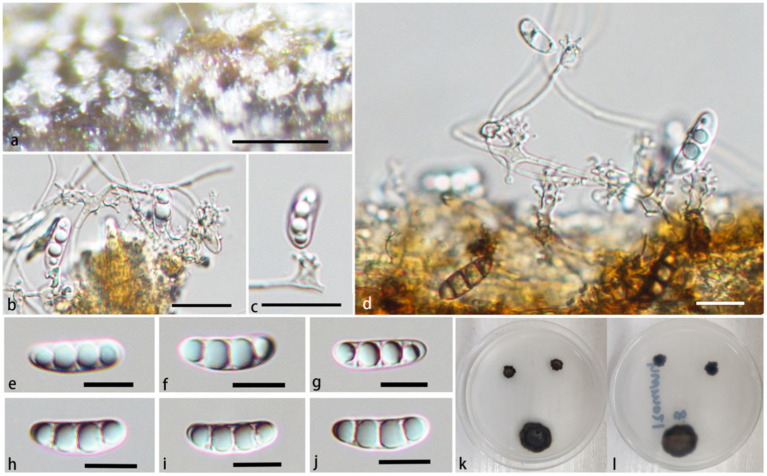
*Pleurothecium jiangxiense* (HFJAU 10278, holotype). **(A)** Colonies on submerged wood. **(B–D)** Conidiophores and conidiogenous cells. **(E–J)** Conidia. **(K,L)** Cultures on PDA medium (**K** from above; **L** from below). Scale bars: **(A)** = 100 μm. **(B–J)** = 12.5 μm.

In addition, *P. jiangxiense* have similar conidiophores and conidiogenous cells to genus *Neta*, but its denticles are denser than *Neta*. *Pleurothecium jiangxiense* have 3-septate conidia, while species of *Neta* often have 1-septate conidia (Figure 6).

## Discussion

*Pleurotheciaceae* members are mainly distributed in the tropics and subtropics, with a few in central and southern Europe, such as *P. semifecundum*, *P. recurvatum*, *Pla. erumpens*, and *Pla. rivularia* (Réblová et al., 2012; Réblová et al., 2020). Most species of *Pleurotheciella* and *Pleurothecium* have been reported in aquatic habitat from Yunnan province, China, and Thailand (Luo et al., 2018). Inaddition, two species, *Pla. nilotica* and *Pla. obovoideum*, have been reported in Japan and and Egypt, respectively, (Arzanlou et al., 2007; Abdel-Aziz et al., 2020). Currently there are no reports of *Pleurotheciella* or *Pleurothecium* from Jiangxi Province, China.

Ten strains including four new species introduced in this paper are all from the freshwater environment in Jiangxi Province. *Pleurotheciaceae* species observed mostly from submerged decaying woods (Luo et al., 2018; Réblová et al., 2020). The 10 strains examined in this study were all recovered from decaying wood within a freshwater ecosystem. These strains were observed and isolated from the wood over a period of time. Upon observing a substantial collection of specimens, no sexual reproductive patterns have been identified. Consequently, it appears that species within the genera *Pleurotheciella* and *Pleurothecium* may predominantly exist in an asexual form within their natural habitat.

According to phylogenetic analyses, species of *Saprodesmium* and *Neomonodictys* have longer genetic distance from other species of *Pleurotheciaceae*. This observation underscores the need for additional research and discovery to enhance the taxonomic classification within the *Pleurotheciaceae*. *Sterigmatobotrys* has not been definitively identified as a genus within the *Pleurotheciaceae* family, but *Sterigmatobotrys* groups together with species of *Pleurotheciaceae* with a strong statistical support (Figure 1). Previously, *P. obovoideum* was reported with a separate phylogenetic tree not fully integrated into other species sequences of *Pleurotheciaceae*, which have some problems in its taxonomic position (Arzanlou et al., 2007). According to phylogenetic analysis, we adjusted the taxonomic position of *Pleurothecium obovoideum* to *Neomonodictys obovoideum* by its strong clade support (PP/BS = 1.00/100) clades with *Neomonodictys aquatica* and *Neomonodictys muriformis.*

Previously, all asexual morphs of *Pleurotheciella* collected from natural environment have macronematous conidiophores, which distinguished from *Dactylaria species* (Luo et al., 2018). Previous studies have shown that *Pleurotheciella* species have macronematous conidiophores in natural environment, our specimens further confirm this phenomenon. At present, there are still five *Pleurothecium* species without molecular data, other species of *Pleurothecium* form a well-supported monophyletic clade in the *Pleurotheciaceae*. Morphologically, *Pleurothecium* species are similar to *Neta* in conidiogenous cell and conidiophores, but *Pleurothecium* have denser denticles and more obvious cylindrical conidiogenous site and 3-septate conidia, which significantly distinguished from *Neta*.

## Data Availability

The datasets presented in this study can be found in online repositories. The names of the repository/repositories and accession number(s) can be found in the article/Supplementary material.

## Footnotes

^1^ http://inocybe.info/_userdata/ruler/PhotoRuler.html

^2^ http://www.ncbi.nlm.nih.gov/
